# Experimental Heart Failure Models and Their Pathophysiological Characterization

**DOI:** 10.1155/2016/2538263

**Published:** 2016-01-13

**Authors:** Peter Moritz Becher, Bodh I. Jugdutt, John Baugh, Bastian Schmack

**Affiliations:** ^1^Department of General and Interventional Cardiology, University Heart Center Eppendorf, 20246 Hamburg, Germany; ^2^Division of Cardiology, Department of Medicine, Mazankowski Alberta Heart Institute, University of Alberta and Hospitals, 8440-112 Street, Edmonton, AB, Canada T6G 2B7; ^3^Conway Institute of Biomolecular and Biomedical Research, School of Medicine and Medical Science, University College Dublin, Belfield, Dublin 4, Ireland; ^4^Department of Cardiac Surgery, Heart and Marfan Center, University of Heidelberg, 69120 Heidelberg, Germany

Heart failure (HF) is a leading cause of morbidity and mortality in the Western world. Despite implementation of current recommended therapies for the treatment of the HF syndrome [1, 2], prevalence, mortality, and costs associated with HF are rising. Expansion of our aging population with high prevalence of such comorbidities as coronary artery disease, myocardial infarction, hypertension, diabetes, and obesity that predispose patients to this complex syndrome is expected to increase HF prevalence even further in the future. Current treatment options and strategies [1, 2] predominantly slow the progression of the HF syndrome. There is a need to develop novel preventative and reparative therapy options. However, development of these novel HF therapies requires testing of the putative therapeutic strategies in appropriate HF animal models [3]. The primary goal of experimental animal HF models is to simplify an indeed complex syndrome into manageable research questions in reproducible settings. The ultimate goal is to elucidate pathophysiological mechanisms and identify key pathways that can be targeted for developing therapies that can be tested in appropriate translational animal models before evaluation in clinical trials for prevention and improving outcome of HF in humans. While there are multiple causes of HF, the dominant ones are valvular heart disease, dilated cardiomyopathies, hypertensive heart disease, and restrictive cardiomyopathies.

The two major clinical phenotypes are HF with reduced ejection fraction (HFrEF) and HF with preserved ejection fraction (HFpEF) [4]. Right-sided HF and pulmonary hypertension also play important roles in the HF phenotype. Various small and large animal models have been used to induce HF [3], including volume and pressure overload, rapid pacing, myocardial infarction with or without coronary reperfusion, coronary embolization, cardiotoxic drugs, or genetic variations (in small animals).

The number of HF patients is increasing owing to the deficiency of therapeutic approaches to treat this population of patients. A discourse between clinicians and scientists seems to be essential to develop novel experimental animal models of HF that accurately imitate the complex clinical syndrome of HF.

During the past decades, the use of experimental animal models to examine complex cardiovascular pathophysiology has been confirmed to be irreplaceable in this field [5]. As a result of basic and translational experiments in small animal models, our understanding of the pathophysiology of HF and its treatment has advanced significantly.

In addition, the ability to manipulate the mouse genome has simplified a particularly important approach to detect novel therapeutic targets, offering a significant approach to explore the mechanisms underlying development and progression of the HF syndrome [5].

Moreover, the adaptation of present experimental animal models will be required to entirely translate scientific findings into new drugs and therapeutic approaches. Future animal models of HF will hopefully give mechanistic insights that could lead to novel options of therapies.

Experimental animal models of HF, as opposed to isolated organ and/or cell preparations, do empower examination of the physiological effects of cardiac functioning, which are of excessive significance in the HF phenotype [3].

Moreover, manipulation of the mouse and rat genomes has allowed significant mechanistic insights into different HF phenotypes in humans.

Although mice are relatively economical and suitable, substantial differences exist between mouse and human heart physiology and especially during development and/or progression of HF [5]. For instance, mouse hearts are very small and do beat very fast (400–600 beats per minute) [6] compared with human hearts (60–90 beats per minute). These dissimilarities lead to important alterations in calcium handling and ion currents between the two species.

Mutations in the giant sarcomeric protein titin (Ttn) are a major cause for inherited forms of dilated cardiomyopathy (DCM). In this issue, Q. Zhou et al. investigated a pattern of DCM that can be induced by TAC-mediated pressure overload in a Ttn-truncated mouse model. This model expands the resource of cardiac disease models, adding a valuable tool to understand cardiac pathophysiological remodeling processes and to develop therapeutic approaches to combat HF.

J. Talavera et al. examined an improved protocol in the rabbit model of anthracycline-induced cardiomyopathy. Current protocols of anthracycline-induced cardiomyopathy in rabbits had disadvantages for long-term studies such as high premature mortality and toxicities (e.g., nephrotoxicity). With the aim of obtaining a more appropriate protocol for this kind of research, the researchers developed a shortened protocol of anthracycline-induced cardiomyopathy using daunorubicin of 4 mg/kg/week over a period of six weeks resulting in high incidence of overt dilated cardiomyopathy with more stable signs of congestive HF, associated with reduced systemic compromise and very low premature mortality. This refined model in rabbits can be very useful for long-term studies aimed at evaluation of the functional effects of novel therapies for HF in anthracycline-induced cardiomyopathy.

E. Roussel et al. performed a gene expression profile of the model of chronic volume overload in rats with severe aortic valve regurgitation (AR) over a period of 9 months. The investigators focused on the study of genes associated with myocardial energetics in that model. Their results displayed that the myocardium with chronic volume overload sustained significant metabolic stress and developed important energetics adaptations. Clinicians currently follow those patients without any intervention for a good number of years, simply waiting for the left ventricle to become too dilated, for the occurrence of symptoms, or until systolic function begins to fall. The findings of E. Roussel et al. in this issue suggest that those hearts develop severe metabolic abnormalities even when systolic function appears to be preserved and that intervention then can limit the dilation and metabolic abnormalities. Focusing on myocardial metabolism by various interventions such as targeted drugs, specific diets, or exercise may help this metabolically stressed myocardium to improve its energy production and may prolong the pre-HF state significantly. However, E. Roussel et al. have observed that treatment with fenofibrate, a PPAR*α*-agonist, normalized both fatty acid and glucose uptakes while reducing left ventricular dilation caused by AR.

Right ventricular (RV) dysfunction due to chronic pressure overload is a common feature of congenital heart diseases. Here, M. Hirata et al. propose an improved pulmonary artery (PA) banding procedure using a half-closed clip (PAC) instead of partial ligation in the rat model of RV dysfunction secondary to chronic pressure overload.

T.-H. Chen et al. used the conditional HSP60 transgenic mouse model to demonstrate neonatal death and HF with transgenic HSP60 expression, likely due to atrial septal defects, increased apoptosis, and myocyte degeneration and other cardiac developmental defects. Since this mitochondrial heat shock protein is essential for maintaining life, they suggest that the model can be useful for addressing other important biological questions about HSP60.

There exists a solid body of evidence that the carotid body (CB) chemoreflex is relevant during the progression of chronic HF. Here, D. C. Andrade et al. reviewed the relevance of CB chemoreflex during the progression of HF. The authors emphasize that several HF experimental models also display a heightened CB chemoreflex drive which correlates positively with the severity of the disease. Moreover, recent exciting studies indicate that ablation of the CB chemoreceptors not only improves autonomic function and reduces disordered breathing patterns in experimental CHF, but also improves survival.

These findings raise the question of whether the CB chemoreflex should be tested in all types of HF (i.e., HFrEF and HFpEF). To sum up, future studies should discuss the role of the CB in the progression of autonomic imbalance and disordered breathing patterns in nonsystolic chronic HF (HFpEF).

We hope that this special issue will help readers become familiarized with recent progress regarding experimental heart failure models and their pathophysiological mechanisms.
